# Problem Talk in Adolescence: Temperament and Attachment as Predictors of Co-Rumination Trajectories in Boys and Girls

**DOI:** 10.3390/brainsci11091157

**Published:** 2021-08-31

**Authors:** Margot Bastin, Amy H. Mezulis, Jaclyn T. Aldrich, Guy Bosmans, Sabine Nelis, Filip Raes, Patricia Bijttebier

**Affiliations:** 1 Faculty of Psychology and Educational Sciences, KU Leuven, Tiensestraat 102, 3000 Leuven, Belgium; guy.bosmans@kuleuven.be (G.B.); filip.raes@kuleuven.be (F.R.); patricia.bijttebier@kuleuven.be (P.B.); 2 Adolescent Cognition and Emotion (A.C.E.) Lab, Seattle Pacific University, 3307 3rd Ave W, Seattle, WA 98119, USA; mezulis@spu.edu; 3 Seattle Children’s Hospital, 4800 Sand Point Way NE, Seattle, WA 98105, USA; aldrichj@spu.edu; 4 Thomas More, Molenstraat 8, 2018 Antwerpen, Belgium; sabine.nelis@thomasmore.be

**Keywords:** co-rumination, temperament, attachment, gender, adolescence

## Abstract

Co-rumination has consistently been shown to be maladaptive in the context of emotional well-being. However, not much is known about factors that predict one’s tendency to co-ruminate. The current study investigated temperament, attachment, and gender as predictors of co-rumination trajectories in a sample of 1549 early and middle adolescents from fifth to ninth grade (53.4% girls; *M*_age_ = 12.93). Analyses were performed on four waves of data with one-year intervals using multi-level modeling. First, girls were found to be more likely to co-ruminate. Second, high positive affectivity in boys and girls and high effortful control in boys was related to higher co-rumination. Third, high attachment anxiety and high general trust in the availability and support of a mother were predictive of higher co-rumination levels. High attachment avoidance was negatively related to co-rumination in boys. High positive affectivity in boys and girls and high trust in boys predicted decreases in reported co-rumination levels over time. Results highlight differences between boys and girls in factors that predict the tendency to co-ruminate. The current study adds to the literature by helping to identify factors associated with the development of co-rumination, which is a well-established risk factor of internalizing symptoms. Monitoring youth affected with these vulnerabilities may be recommended for prevention efforts.

## 1. Introduction

In adolescence, peer relationships become a primary source of emotional support [1]. Disclosing personal information to a friend cultivates closeness and satisfies one’s need to belong, thereby contributing to emotional well-being [2]. However, one way of talking about negative experiences with friends that has been shown to be maladaptive for mental health, is co-rumination. Co-rumination is defined as “excessively discussing personal problems within a dyadic relationship” [3] (p. 1830). It is characterized by extensively talking about problems, rehashing problems, speculating about problems, mutual encouragement of problem talk, and dwelling on negative affect [3]. Co-rumination is situated at the intersection of rumination and self-disclosure [4]. 

Extant research shows that co-rumination with friends is associated with both adaptive and maladaptive outcomes. On the one hand, consistent with findings on self-disclosure, co-rumination provides social rewards. Specifically, it is associated with higher and increasing levels of friendship quality and closeness [3]. On the other hand, co-rumination also predicts higher concurrent and prospective internalizing symptoms in general [5]; see [6] for a meta-analysis, and higher depressive [7] and anxiety [8,9] symptoms in particular. Furthermore, co-rumination has been found to predict the lifetime history of clinical depression [10], as well as the onset, severity, and duration of future depressive episodes, including first onsets [11] and has been shown to play a role in the increase of interpersonal peer stressors [5,12]. 

Given extensive research linking co-rumination to emotional problems and stressors among adolescents, it is important to understand what factors make individuals prone to this tendency to co-ruminate. However, research on this is very scarce. One study has examined the concurrent relationship of supportive and non-supportive maternal responses to adolescents’ negative emotions with adolescents’ levels of co-rumination [13]. Positive associations were established between supportive maternal responses and co-rumination [13]. The purpose of the current study was to further gain insight into aspects that may render individuals vulnerable to co-ruminate. To gain insight into contributing factors at more than just one level, we studied both individual characteristics of the participants and factors situated in the interpersonal context. Specifically, we investigated temperament (i.e., individual differences in affective-motivational reactivity and self-regulation), attachment (i.e., the representations of relationships between child and parent), and gender as predictors of co-rumination trajectories. Research questions were addressed in a multi-wave sample of early and middle adolescents, as adolescence is characterized by an increased salience of friendships [1] and as this developmental phase has been shown key in understanding peer processes [14].

### 1.1. Temperament

Temperament is conceptualized as constitutionally based, relatively stable individual differences in emotional, behavioral, and attentional reactivity and self-regulation [15]. The reactivity aspect of temperament reflects individual differences in emotional arousability and is typically subdivided into negative affectivity (NA) and positive affectivity (PA) [16]. NA is characterized by high levels of negative affect, low approach motivation, high and intense reactivity to stimuli, and low adaptability [15]. Individuals high in NA typically avoid novel situations, experience high subjective distress in such situations, and typically feel afraid, agitated, or frustrated [17]. PA, on the other hand, is characterized by high levels of positive affect, high sociability, high adaptability, and a high tendency to approach stimuli [15]. These individuals have a greater tendency to approach a new environment and they typically feel active, happy, and enthusiastic [17]. 

Multiple studies have demonstrated that NA predicts cognitive vulnerabilities, such as rumination [18,19,20]. For instance, NA has been shown to be predictive of higher weekly levels of rumination in a sample of young adults [18]. Relations between NA and rumination have also been established in the long term, with Mezulis and colleagues [21] finding that NA at age 1 predicted levels of rumination at age 13. As rumination is considered the intrapersonal variant of co-rumination, NA is likely related to increased focusing on and dwelling on negative feelings in conversations within dyads (i.e., co-rumination) as well. PA, on the other hand, has been put forward as being of great significance for strengthening affective bonds, for providing a basis for relationships characterized with trust, and for helping people to perceive others as more trustworthy and reliable [22,23]. Further, it has been shown to provide a foundation to form a sense of fellowship and to stimulate individuals to disclose more personal information to whom they like [24]. For example, Yu and colleagues [23] and Forgas [25] showed that university students who experienced higher levels of PA tended to disclose more intimate information about themselves to others. Thus, PA may be predictive of higher co-rumination, given its positive associations with social bonding and self-disclosure. 

The self-regulatory aspect of temperament comprises effortful processes that enable individuals to modulate their emotional reactivity by facilitating or inhibiting affective and behavioral responses. Rothbart [26] introduced the concept of effortful control (EC) to refer to the capacity to self-regulate predominant behavioral and emotional responses. Individuals low in EC are less able to actively suppress or control their NA and/or PA, and they may lack inhibiting or activating behaviors, or the ability to voluntarily shift and focus the attention [16]. As such, previous research has shown that low levels of EC strengthen the relation between reactivity and negative cognitive style (i.e., interaction effect). For example, rumination has been established as a mediator of the relationship between NA and depressive symptoms only in adolescents with low levels of EC [27]. Similarly, low EC may prevent adolescents to keep them from co-ruminating when faced with problems and negative emotions. 

### 1.2. Attachment

According to attachment theory, individuals develop patterns of relational expectations, emotions, and behaviors that result from the internalization of a particular history of care-related experiences [28,29]. The internal working models that reflect those internalized attachment experiences form the blueprint for the development of relationships and interactions with peers later in life [30]. 

The quality of early attachment development is conditional upon the levels of parental support experienced by the child [28]. Specifically, children who experience high trust in the availability of a parent (i.e., securely attached children) view their caregivers as caring, supportive, responsive, and available [31]. When trust in parental support and in the availability of parents is low, two insecure attachment dimensions are described: attachment anxiety and attachment avoidance. Individuals high in attachment anxiety seek support during distress, but continuously fear abandonment and rejection by attachment figures; those high in attachment avoidance, by contrast, avoid support seeking during distress and only rely on themselves [32]. As individual differences in attachment are continuously distributed rather than categorically, attachment is generally described as dimensional [33]. 

(Lack of) trust in the availability of a caregiver predicts which strategies children rely on to regulate their emotions [34,35]. Specifically, youth who have more trust in the support and the availability of attachment figures are more likely to rely on support seeking strategies to cope with distress and to prefer open communication when distressed [35]. It is possible that these individuals are not only more prone to self-disclose in general, but they may also engage in greater problem focused communication (i.e., co-rumination). More anxiously attached children tend to hyperactivate negative affect in order to elicit support and care from others [36]. They have more difficulty disengaging from negative experiences and regulating their emotions. As such, they are more likely to ruminate [37,38]; but see [39]. Individuals higher in attachment avoidance, on the other hand, are more likely to avoid, suppress, or deactivate negative affect and minimize emotional displays rather than openly disclose to others [40]. They tend to create emotional distance and aim to avoid dependence on others [35]. Associations with rumination have been established before; but findings are mixed [38,41,42]. Based on these associations with emotion regulation, it can be expected that more anxiously attached individuals will display more co-rumination as attachment anxiety reflects both the desire for interpersonal contact with others and the vulnerability to ruminate on negative affect. In contrast, as attachment avoidance is associated with discomfort with closeness and intimacy, more avoidant attached youth may be expected to co-ruminate less. 

### 1.3. The Role of Gender

Literature thus far has shown gender differences in co-rumination, with girls reporting higher co-rumination levels compared to boys, both in childhood and in adolescence [4,5]. A plausible explanation is that relations between the proposed predictors of co-rumination and co-rumination itself are stronger for girls than for boys. For example, differential socialization practices of boys’ and girls’ responses to affect may result in a stronger relation between temperament reactivity and certain emotion regulation strategies among girls compared to boys [43]. Research has shown that when children express role-consistent emotions, parents provide more attention to the child [44] and, as such, they are more accepting of sadness and anxiety in girls compared to anger in boys [45]. Girls are thus encouraged to talk about negative feelings (i.e., link with co-rumination) and to seek proximity when they are feeling sad, whereas boys are encouraged to keep their feelings to themselves [46].

Associations between attachment and co-rumination may differ for boys and girls as well. As parents more generally discourage emotion expression in boys compared to girls and as parents talk more about feelings of sadness with daughters than with sons [47], it can be expected that more secure attachment in youth (i.e., higher levels of trust) will be more strongly associated with co-rumination in girls compared to boys. Also studies with a focus on associations between insecure attachment and emotion expression have pointed towards gender differences in such associations. In a study with newlywed couples, for instance, higher attachment avoidance in women was found to be associated with a greater willingness to tolerate negative emotions, whereas the opposite was true for men [48]. The authors hypothesize that more avoidantly attached men may be less likely to use their emotional state as a source of information to guide their behavior compared to women [48]. This may result in a stronger negative association between attachment avoidance and problem talk in boys. 

### 1.4. The Current Study

With the current study, we aimed to gain insight into predictors of co-rumination. The first predictor examined was temperament. We expected NA to be a positive predictor of co-rumination, as previous studies have shown NA to be an antecedent of intrapersonal rumination [18]. In addition, the tendency to experience negative affect more easily (i.e., NA) might be related to a greater discussion of these negative feelings that arise. We expected PA to be a positive predictor of co-rumination as well, given previous research on the positive relationship between PA and self-disclosure [24]. To acknowledge the complexity of temperament, both PA and NA were examined in combination with adolescents’ regulatory capacities (i.e., EC). Associations with co-rumination were expected to be especially strong for individuals low in EC and high in NA [27]. 

Our second aim was to investigate attachment as a predictor of co-rumination. The study of attachment was carried out in two ways. First, we investigated whether general trust predicted engagement in co-rumination, hypothesizing that higher levels of trust would make youth more prone to co-ruminate with a friend. Specifically, as higher levels of trust are associated with approaching important attachment figures [49], it was expected to relate to higher levels of both adaptive and maladaptive forms of self-disclosure. Second, we evaluated the unique predictive value of the two insecure attachment dimensions, i.e., attachment anxiety and attachment avoidance. We hypothesized that the association between co-rumination and attachment avoidance would be negative, as higher attachment avoidance is characterized by greater interpersonal distance and suppression of feelings [40]. For attachment anxiety, two alternative hypotheses may be put forward. On the one hand, as adolescents high in attachment anxiety have been found to express more dependent behavior and to reach out [36], they may also be expected to engage in more co-rumination. On the other hand, out of the conceptualization that higher attachment anxiety reflects lower levels of attachment security (i.e., lower trust), it can also be expected that attachment anxiety, like attachment avoidance, would relate to lower levels of co-rumination. 

In these two research questions, the moderating role of gender was taken into account. As mentioned previously, we expected the relationship between temperament and co-rumination to be stronger for girls. We further hypothesized a stronger positive relationship for girls compared to boys between co-rumination and trust in the availability of a parent and between co-rumination and attachment anxiety. For attachment avoidance, we expected a stronger negative association with co-rumination for boys. 

To gain insight into predictors of co-rumination, we examined associations with trajectories of co-rumination over a 3-year interval. This way, temperament, attachment, and gender could be studied not only as predictors of initial co-rumination levels, but also of changes in levels over time. Furthermore, to obtain a stringent test for the role of temperament and attachment, we examined our research questions while covarying for related constructs of co-rumination, being brooding rumination and depressive symptoms [3,11]. 

## 2. Materials and Methods

### 2.1. Participants 

For the current study, 28 schools were randomly selected from an exhaustive list of all schools in Flanders—the Dutch-speaking part of Belgium. Principals were contacted by a letter and a few weeks later by a phone call. If the school boards were willing to participate in the study, a visit to the school was scheduled. This was the case for seven schools. Next, consent forms were distributed to parents of 1733 adolescents from fifth to ninth grade. A total of 86 parents did not give their child permission to participate. At the first assessment, 90 pupils were absent (mostly due to illness) and data of seven pupils could not be used because of random patterns in responses. One pupil did not want to participate. The final sample consisted of 1549 early adolescents, who all gave assent. Participants’ age ranged from 9 to 17 years old (*M*  =  12.93; *SD*  =  1.46) and 53.4% were girls. Initial CDI scores were in the clinically significant range for 16.90% of the participants (i.e., score ≥ 16 [50]). Information on nationality was collected at the second measurement wave. Of all participants present at the second assessment, 93.0% reported the Belgian nationality, 2.4% the Dutch nationality, and the other 4.6% had a different nationality (e.g., Afghan, German, Moroccan, Polish, Portuguese, Russian). The population was predominantly Caucasian and can be considered as representative of the Belgian population in terms of SES. A total of 19.5% of the participants further reported that their parents had gotten divorced and 1.8% reported that at least one of their parents had deceased. At the conclusion of the study, the distribution of the participants within different schooling branches included 79.3% in general secondary education, 7.5% in vocational education, and 13.2% in technical education.

Of all participants present at Time 1, 48.87% completed measurements at all four assessments, 16.14% completed three out of the four assessments, 15.24% completed two assessments, and 19.75% completed one assessment. Between the 1007 pupils who were present at three or four assessments versus the 542 pupils who completed only one or two assessments, no significant baseline differences were found for co-rumination, *t*(1028.77) = 0.96, *p* = 0.34. However, the group that participated at only one or two assessments reported significantly higher depressive symptoms, *t*(958.02)  =  6.93, *p* <  0.001, higher brooding, *t*(1038.64)  =  2.47, *p* = 0.01, higher NA, *t*(1012.55)  =  2.80, *p* = 0.01, higher attachment anxiety, *t*(926.13)  =  4.02, *p*  < 0.001, and higher attachment avoidance, *t*(1539) = 2.20, *p* = 0.03. Further, this group reported lower EC, *t*(953.16)  =  −6.56, *p*  < 0.001, lower PA, *t*(1005.26)  =  −2.76, *p* = 0.01, and lower trust in the support and availability of the mother, *t*(937.98)  =  4.17, *p*  < 0.001. We observed a significant gender difference, *χ*2(1)  =  4.73, *p* = 0.03, with more girls than boys in the group that was present during at least three assessments. 

### 2.2. Measures

The *Co-Rumination Questionnaire* (CRQ) is a 27-item self-report questionnaire that measures the extent to which individuals typically co-ruminate with their closest, same-gender friend [3]. Items are rated on a 5-point Likert scale, ranging from 1 (not at all true) to 5 (really true). Nine different content areas are covered, including (a) frequency of discussing problems, (b) discussing problems instead of engaging in other activities, (c) friend encouraging discussion of problems, (d) target child encouraging friend to discuss problems, (e) discussing the same problem repeatedly, (f) speculation about causes, (g) speculation about consequences, (h) speculation about parts of the problem that are not understood, and (i) focusing on negative feelings. An example item is “When we talk about a problem that one of us has, we try to figure out every one of the bad things that might happen because of the problem”. For the present study, the shortened 9-item version, with one item for each of the nine content areas, was used [5]. Previous research has shown that this version is reliable [5,11]. The total score was calculated as the mean of the nine items. Cronbach alpha in the current study is 0.87. 

The *Positive Affect and Negative Affect Scales* (PANAS) assesses both NA and PA. Each scale consists of 10 negative and 10 positive emotional states (e.g., “ashamed”, “guilty”, “strong”, “enthusiastic”) [51]. Participants were asked to indicate how often they feel as described in daily life, using a 5 point-Likert scale, ranging from 1 (very slightly/not at all) to 5 (extremely). The PANAS was originally designed for adults, but has demonstrated satisfactory reliability and good convergent, construct, and discriminant validity in adolescent samples [52]. Cronbach alpha in the current study is 0.81 and 0.84, for the PA and NA scale respectively. 

The *Effortful Control Scale* (ECS) is a 24-item self-report questionnaire that measures attentional control, activation control and inhibitory control as aspects of effortful control [53]. Items are rated on a 5-point Likert scale, ranging from 1 (not at all like me) to 5 (very much like me). Two subscale scores can be derived (i.e., Persistence/Low distractibility versus Impulsivity). The internal consistency and construct validity of the ECS has been previously supported [54]. For the current study, only the Persistence items of the ECS were included (e.g., “I start many things that I don’t finish”). In previous studies, this subscale demonstrated stronger correlations with other measures of effortful control compared to the Impulsivity scale [54]. Also, there is some disagreement as to whether more automatic types of control, like impulsivity, adequately measure effortful control [55]. Cronbach alpha in the current study is 0.84.

The “Trust” subscale of the *People in My Life Questionnaire* (PIML) is used to assess trust in maternal support and availability [56]. The subscale consists of 10 items. Items are rated on a 4-point Likert scale, ranging from 1 (almost never true) to 4 (almost always true). An example item is “My mother respects my feelings”. Concurrent and convergent validity of the trust-scale has been established previously [57]. Cronbach alpha in the current study is 0.94.

The *Experiences in Close Relationships Scale—Revised Child version* (ECR-R) is a 36-item self-report questionnaire assessing both anxious and avoidant attachment [58,59]. Items are rated on a 7-point Likert scale, ranging from 1 (strongly disagree) to 7 (strongly agree). For the current study, the shortened 12-item version was used [59]. Only items relating to the mother were included, resulting in six avoidant attachment (e.g., “I prefer not to show to my mother how I feel deep down”) and six anxious attachment items (e.g., “I worry that my mother does not really love me”). The ECR-RC has been shown to be a reliable and valid instrument [59]. Cronbach alpha in the current study is 0.85 and 0.81, for attachment anxiety and attachment avoidance, respectively.

The *Children’s Depression Inventory* (CDI) is a 27-item self-report questionnaire that measures cognitive, affective, and behavioral symptoms of depression over the past two weeks [60]. Items are rated on a 3-point rating scale (0–2) with higher scores representing more severe symptomatology. Total scores on the CDI are a sum of all items. An example item is “I am sad once in a while/I am sad many times/I am sad all the time”. The CDI has been shown to be reliable and valid and discriminates individuals with major depressive disorders from nondepressed individuals [60]. Cronbach alpha in the current study is 0.86.

The “brooding” subscale of the *Children’s Response Styles Questionnaire—extended version* (CRSQ) consists of 5 items and measures brooding rumination in response to sadness [54,61]. Items are rated on a 4-point Likert scale, ranging from 1 (almost never) to 4 (almost always). An example item is “Thinking ‘What have I done to deserve this?”. Scores range from 5 to 20, with higher scores indicating a greater tendency to brood. Cronbach alpha in this study is 0.79.

### 2.3. Procedure

All adolescents and parents in the participating schools received letters explaining the purpose of the study, inviting them to join the study and asking for parental permission. Pupils who received permission to participate were asked to sign consent forms before the start of the study. Questionnaires were filled out collectively during school hours. The four assessments, with one-year intervals, followed the same procedure. The coordinator of the study and at least one master’s student in psychology were present to answer questions. Ethical approval was obtained from the local research Ethics Committee. 

### 2.4. Data Analyses

Multi-level modeling in HLM 8.1 was used to analyze data. It allows for missing data for participants not present at all assessments by including all data in the analyses, resulting in optimal use of data. It furthermore accounts for baseline differences in variables by including the variables in equations.

HLM identifies regression equations which describe trajectories of individuals over time by calculating an intercept and a slope. The intercept refers to the individuals’ initial level on the dependent variable, whereas the slope describes changes in the dependent variable over time. We predicted both intercepts and slopes of trajectories. The size of the current sample was lower than typically recommended for models including quadratic effects, especially for models run separately in boys and girls [62]. Furthermore, possible quadratic effects are most reliable when at least five points of data are available [62]. Because of these reasons, we only investigated linear change.

HLM calculates equations at two levels. Level 1 equations capture each individual’s trajectory of co-rumination as a function of time (i.e., four waves of data in the current study). Level 2 equations examine the differences between the Level 1 trajectories of the individuals as a function of the variables included in Level 2. In our study, Level 2 variables include gender, baseline temperament, baseline attachment, the temperament × gender interactions, and the attachment × gender interactions. This longitudinal approach thus indicates within-participants changes in co-rumination levels over time, while also taking into account variation between individuals as a function of differences in the included predictors. Because previous research consistently established associations of co-rumination with brooding rumination and depressive symptoms, both variables were included as covariates in analyses. Given significant associations between age and study variables (see below), age was included as a covariate as well. 

Separate models were run for temperament and attachment. Gender was coded −1 (boys) and 1 (girls) and the scores on all other predictors and covariates were standardized, except for age, which was expressed in months. We used full maximum-likelihood estimation and iterations were set at 1000. Time points were coded as 0, 1, 2, and 3. 

## 3. Results

### 3.1. Descriptive Statistics

Descriptive statistics and intercorrelations between all baseline variables and between baseline variables and co-rumination at all four time points are reported in Table 1; Table 2. Medium to large correlations were found between co-rumination measures at all time points. NA was significantly and positively correlated with co-rumination at all time points and PA showed (small) positive associations with co-rumination scores at T1 and T2. No significant associations were established between co-rumination and EC or between co-rumination and trust. Weak associations with co-rumination scores were found for attachment anxiety (positive association) and attachment avoidance (negative association) across the four assessments. Age was weakly associated with almost all variables (all positive correlations, except for negative correlations for EC and trust), with the exception of non-significant associations with PA and attachment anxiety. 

Some significant gender differences were observed. Girls reported significantly higher levels of NA and co-rumination than boys, whereas boys reported higher levels of PA and attachment avoidance than girls.

### 3.2. Co-Rumination Trajectories over Time 

In the first step, co-rumination trajectories were examined as a function of time only. The intercept was significantly different from zero (coefficient = 3.17, *t* = 175.00, *p* < 0.001). There was no significant effect of time, indicating a rather flat slope for the total group (coefficient = 0.01, *t* = 1.32, *p* = 0.19). In the next step, gender and the three covariates (brooding, depressive symptoms, and age; see Table 3) were added to the model. Results indicated that initial co-rumination levels did vary as a function of gender, age, and brooding. Specifically, the co-rumination intercept started at a higher level for girls, for individuals higher in brooding, and for older adolescents. Levels of co-rumination for the entire sample did change differently as a function of gender, with slopes being more negative for boys than for girls. Covariates were kept in the model if *p*-values were ≤ 0.10. As a result, all covariates were retained to predict the intercept of co-rumination trajectories, including depressive symptoms with *p* = 0.051, whereas only gender was retained as a predictor of the slope.

### 3.3. Temperament as a Predictor of Co-Rumination Trajectories

To test the hypothesis that temperament would predict co-rumination trajectories, all temperament variables were included as predictors of intercept and slope. Specifically, predictors included (a) the affectivity components NA and PA, (b) the self-regulatory component EC, and (c) interactions between (a) and (b). In this model (i.e., a model without including interactions with gender), gender (coefficient = 0.24, *t* = 14.13, *p* < 0.001), EC (coefficient = 0.05, *t* = 2.48, *p* = 0.01), and PA (coefficient = 0.12, *t* = 6.30, *p* < 0.001) uniquely predicted the intercept of co-rumination trajectories with coefficients being positive. Slopes of co-rumination trajectories were predicted by PA, with slopes declining for individuals high on PA (coefficient = −0.03, *t* = −3.51, *p* < 0.001); the other predictors were not significant. 

Next, two-way and three-way interactions with gender were added as Level-2 predictors. Results are presented in Table 4. On top of the previously mentioned main findings on slope and intercept, significant interactions were found between gender and NA and between gender and EC in predicting the intercept. To investigate this interaction, multilevel models were run for boys and girls separately. These post-hoc examinations indicated that the positive association between EC and the intercept of co-rumination was significant in boys (coefficient = 0.11, *t* = 3.23, *p* = 0.001); not in girls (coefficient = 0.00, *t* = 0.17, *p* = 0.86). For NA, associations with co-rumination intercepts differed between both, yet coefficients were non-significant in both groups separately, i.e., negative (non-significant) coefficient for girls = −0.04, *t* = −1.43, *p* = 0.15 and a positive (non-significant) coefficient for boys = 0.04, *t* = 1.07, *p* = 0.29. No significant interactions were found predicting the slope of co-rumination trajectories.

### 3.4. Attachment as a Predictor of Co-Rumination Trajectories

To test our second main hypothesis, similar HLM analyses were conducted with attachment variables predicting both intercept and slope of co-rumination trajectories above and beyond gender and the covariates. Separate models were run to evaluate trust in the availability of a parent and for the two insecure dimensions (i.e., attachment anxiety and attachment avoidance). (By means of sensitivity analyses, an additional model was run including trust, attachment anxiety, attachment avoidance, and interactions with gender all together in one model. Values of coefficients differed slightly, yet all conclusions based on significance remained the same).

#### 3.4.1. Trust 

First, in the model including the main association with trust (i.e., no interactions with gender added), trust in the availability of the mother predicted both the intercept (coefficient = 0.06, *t* = 2.94, *p* = 0.003) and the slope (coefficient = −0.02, *t* = −2.14, *p* = 0.03) of co-rumination trajectories, with individuals high in trust showing higher initial co-rumination levels and decreasing levels over time. Gender predicted intercept (coefficient = 0.23, *t* = 13.70, *p* < 0.001) and slope (coefficient = 0.02, *t* = 2.01, *p* = 0.05). 

Second, the two-way interaction with gender was added as a Level-2 predictor. Results are shown in Table 5. Additionally to the previously mentioned association, an interaction between trust and gender was found in the prediction of the slope of co-rumination trajectories. Post-hoc analyses showed significant associations with slopes of trajectories for boys (coefficient = −0.04, *t* = −2.62, *p* = 0.01), not for girls (coefficient = −0.01, *t* = −0.55, *p* = 0.58). Gender was no significant predictor of the slope in this model including interactions.

#### 3.4.2. Attachment Avoidance and Attachment Anxiety

In the model including only the main effects, we established significant findings for gender (coefficient = 0.23, *t* = 13.49, *p* < 0.001), attachment anxiety (coefficient = 0.04, *t* = 2.11, *p* = 0.04), and attachment avoidance (coefficient = −0.08, *t* = −4.38, *p* < 0.001) on the intercept. Gender (coefficient = 0.02, *t* = 2.11, *p* = 0.04) and attachment avoidance (coefficient = 0.02, *t* = 1.97, *p* = 0.049) significantly predicted the slope of co-rumination trajectories. 

Second, two-way interactions with gender were added as Level-2 predictors. Results are shown in Table 6. The interaction between gender and attachment avoidance was found to predict the intercept of co-rumination trajectories. To examine this interaction, multilevel models were run for boys and girls separately. The association between attachment avoidance and the intercept was significantly negative in boys (coefficient = −0.15, *t* = −4.90, *p* < 0.001), and not significant in girls (coefficient = −0.04, *t* = −1.79, *p* = 0.07). The significance level of attachment avoidance on the slope of co-rumination trajectories went from 0.049 to 0.055, thereby going from significant to nonsignificant. 

(1). Thanks to an anonymous reviewer, all analyses were reconducted to investigate the influence of age. First, models were run without including age as a covariate. Almost all findings remained the same, with following exceptions: In the model investigating temperament as a predictor, both EC and Gender × NA became marginal significant predictors of the intercept of co-rumination trajectories, estimate(SE) = 0.04(.02), *p* = 0.051 and −0.04(0.02), *p* = 0.075, respectively. In the model investigating attachment anxiety and attachment avoidance, attachment anxiety as well became a marginally significant predictor of co-rumination intercepts, estimate(SE) = 0.04(0.02), *p* = 0.083. Second, all models were rerun, including the interactions between age and study variables. All significant findings reported in the manuscript remained, and none of the interactions with age predicted intercept nor slope). (2). As weak associations have been established between temperament and attachment (for a meta-analysis, see [63]), we ran an additional model including all study variables to control for potential overlap between both and to gain insight into the robustness of the findings. No different conclusions were drawn based on significance levels, with one exception: Trust was not a significant predictor of intercept nor slope of co-rumination trajectories, and the interaction between trust and gender became marginally significant, estimate(SE) = 0.02(0.01), *p* = 0.08. Attachment avoidance and attachment anxiety may be more robust unique predictors of co-rumination trajectories than trust. It would be interesting for future research to further explore this hypothesis).

## 4. Discussion

Co-rumination repeatedly predicts depression, anxiety, and stress in adolescence [6]. The current study assessed temperament, attachment, and gender as predictors of adolescents’ tendency to co-ruminate to get a better understanding of factors that put individuals at risk to engage in this behavior associated with such outcomes. 

### 4.1. Temperament and Co-Rumination Trajectories

As a first aim, temperament was studied as a predictor of co-rumination trajectories. High PA in boys and girls and high EC in boys were found to relate to higher co-rumination. Co-rumination levels were found to decrease slightly over time for adolescents high in PA. 

Adolescents high in PA were thus found to report higher levels of co-rumination. As individuals high in PA easily self-disclose and view others as more trustworthy [23], it may explain that they also self-disclose more frequently with a negative focus. Results seem to suggest that PA may be predictive of the social ability aspect of co-rumination, with social reward and social bonding acting as factors that encourage co-rumination. We further believe that these individuals might be the ones that have less difficulty getting along with others, thereby increasing their opportunities to self-disclose and co-ruminate. Interestingly and importantly, results indicated that PA related to decreasing levels of co-rumination over the 3-year interval. It is possible that adolescents high in PA learn to rely on more adaptive ways to obtain social reward and closeness. For instance, it is possible that these youth learn to switch from a pervasive negative focus to more problem-focused discussion over time. Research showing that individuals high on PA generally respond more effectively to complex and changing situations and engage in more creative problem solving [64], supports this hypothesis. 

In the current study, NA did not emerge as a significant predictor of co-rumination. This was somewhat unexpected as adolescents high in NA generally experience more intense and chronic negative emotions. Yet, this finding adds to work of Byrd-Craven et al. [65], who found that NA did not predict either self-reported or observed co-rumination. Importantly though, the relationship between NA and co-rumination levels in the current study pointed towards opposite directions for boys and girls, potentially resulting in a zero net finding for the total group. 

Associations between EC and co-rumination had not been expected, as this self-regulatory component is generally understood as a factor strengthening the association between reactivity (i.e., PA and NA) and response styles [20]. However, results showed adolescent boys with high levels of EC to be more prone to co-ruminate, regardless of reactivity levels. Thus, boys engaging in co-rumination seem to do this rather deliberate. For girls, no such relation was established. It appears that boys high in EC are better able to regulate emotional and behavioral reactions to subsequently engage in co-rumination and disclose to a best friend. This may be due to them being generally encouraged by society to distract themselves or to engage in problem-solving and activities when they feel bad, rather than to self-disclose [66]. Our finding is surprising, as EC has previously been shown adaptive in the context of psychopathology and emotion regulation in boys and girls [20]. Yet, this result may further substantiate the rising question whether co-rumination may actually be adaptive for boys. Specifically, Rose et al. [4] found co-rumination to predict internalizing symptoms six months later, yet only in girls. Rose et al. [12] further showed depressive symptoms to predict peer stress only for girls high in co-rumination. The combination of stress experience and low (i.e., as opposed to high) co-rumination in boys has also been found to be associated with higher depressive symptoms [67,68]. It may be interesting for future research to further investigate the adaptive versus maladaptive nature of co-rumination among boys.

Finally, no interactions between self-regulation and affectivity were established. This was somewhat unexpected, as we had hypothesized individuals low in EC and high in NA to report the greatest levels of co-rumination. Although this finding is consistent with some prior studies on the interaction between NA and EC in rumination [19], we hypothesize that the potential differences in the function of EC in boys and girls may have contributed to this lack of finding. 

### 4.2. Attachment and Co-Rumination Trajectories

In the current study, adolescent boys and girls with greater trust in the availability of their mother reported higher levels of co-rumination. Boys high in trust showed decreases in co-rumination levels over time, whereas no such relationship was found for girls. Focusing on the two insecure attachment dimensions, results suggested that individuals high in attachment anxiety reported higher levels of co-rumination, whereas boys high in attachment avoidance displayed lower co-rumination levels. 

At first sight, it may be counter-intuitive that individuals with a more secure attachment style engage in behaviors that are maladaptive in the context of emotional wellbeing. Yet, results are less surprising when considering that individuals experiencing high levels of trust in the availability of attachment figures learn to rely on others and openly communicate about how they feel at a young age [35]. This heightened level of support-seeking and self-disclosure might involve talking about problems and negative topics as well, potentially involving heightened co-rumination. Results further suggest that boys high in trust co-ruminate less over time. We do not have a clear hypothesis as for why this might be the case. It is possible that these results are due to regression to the mean [69]. The decrease could also be interpreted as being consistent with the tend and befriend theory [1], with boys generally turning less towards social connections compared to girls. 

Conform our hypotheses, attachment avoidance was associated with lower initial co-rumination, yet this relationship was only found in boys. As attachment avoidance has been related to individuals suppressing how they feel and a preference for emotional distance from others [36], conversations focusing on personal topics were expected to be scarce. As boys are generally discouraged to self-disclose compared to girls, it is not surprising that the lack in emotional expression that comes with high attachment avoidance is strongest in boys. For girls, it is possible that their female friends still elicit self-disclosure and co-rumination in their conversations, as co-rumination generally occurs more often in female dyads [3]. This could then outweigh the tendency to keep emotions to themselves. 

Adolescents high in attachment anxiety reported higher levels of co-rumination. As high attachment anxiety is accompanied by a strong need for closeness and heightened expression of negative emotions [36], this finding is not surprising. We expect that worries about relationships that come along with attachment anxiety [36] become subject of the conversations within a dyad. Yet, at the same time, combining the findings of general trust with the findings of the two insecure attachment dimensions invites for a reflection on the relationship between both. If we understand high attachment anxiety and high attachment avoidance as two different representations of low trust, negative relationships with co-rumination could have been expected to emerge for both (i.e., higher levels on attachment anxiety and attachment avoidance expected to relate to lower co-rumination). Our findings thus further add to research showing that the specific form of attachment insecurity is important to take into account in the context of emotion regulation [49] and contributes to a fine grained understanding of the role of insecure attachment. We expect that the positive associations of co-rumination with trust and attachment anxiety are driven by different mechanisms. Where the relationship with the former can be expected to be driven by the self-disclosure aspect of co-rumination, the relationship with the latter is expected to be driven by the ruminative process. (Caution may still be warranted when drawing conclusions based on trust specifically, as this variable did not predict co-rumination trajectories in more complex models including both attachment and temperament together, and may thus be a less robust predictor.)

### 4.3. Clinical Implications and Suggestions for Future Research

Worldwide, depression and anxiety disorders constitute a major health issue, with well-documented negative consequences in multiple domains, such as mental and physical health, cognition, and social relations [70]. Understanding antecedents of risks for depression and anxiety (i.e., co-rumination) may contribute to the prevention of internalizing problems. In light of the findings of the current study, we would recommend practitioners to be aware of the characteristics that may relate to a heightened tendency to co-ruminate (e.g., high attachment anxiety) and to monitor youth meeting these vulnerabilities. Adolescents could then be encouraged to limit the negative focus and excessiveness of problem talk or to become aware of the way in which they communicate with friends [71]. Given that co-rumination is also found to occur in relationships characterized by high levels of trust, it will be important to broadly increase awareness of the dangers of excessive problem talk and to encourage the use of adaptive communication styles, like problem solving, a less prolonged focus on negative affect, or expanding to include greater reflection on positive events [72]. Sensitizing this at a young age in conversations with parents may set the example for conversations with friends [73]. As being a girl predicts higher co-rumination, focusing on this subgroup is especially worthwhile. In addition, more research now suggests that co-rumination may be maladaptive for emotional problems in girls in particular [4,67], further supporting the idea that realizing changes in predictors or monitoring youth might be most important in this subgroup. 

The finding that high levels of EC were associated with higher reports of co-rumination in boys, amplifies the question whether co-rumination could be something adaptive for this subgroup. It will be interesting to have more studies looking deeper into differential associations in boys and girls. Gaining insight into the role of EC also has advantages, as EC is easier to tackle in prevention/intervention compared to the reactivity component of temperament (i.e., NA and PA). 

Antecedents other than temperament and attachment may also be considered in future research; with a predominant focus on predictors that can be addressed in therapy and with attention to both distal and proximal factors. For instance, parenting has been acknowledged to be an influential factor in emotional development of youth [74] and has been started to be investigated in the context of co-rumination [13]. Rumination has also been proposed to predict greater co-rumination [3], yet evidence from studies investigating the directionality of relations between rumination and co-rumination does not support this hypothesis [75,76]; but see [77].

### 4.4. Limitations and Strengths

The current study benefits from several strengths, including the examination of a large sample of adolescents, resulting in sufficient power, and the use of multi-level modeling on four waves of data. However, our research also suffers from several shortcomings. First, inflation of relations between our study variables might be present as we made use of self-report. Second, co-rumination trajectories were predicted by variables that were measured at the same time as the first co-rumination measurement. Although temperament and attachment are expected to be stable over time [78,79], including reports in childhood may provide stronger evidence that these risk factors predict co-rumination later in life and it may increase the understanding of temperament and attachment as actual precursors of problem talk in adolescence. Fourth, analyses were not constrained to be stable, reciprocated dyads to measure one’s tendency to co-ruminate. It would be interesting to find out whether the observed relations would replicate in studies including such relations only. Disentanglement of reciprocated friendships might further minimize dependency in data. Finally, the measure of temperament that was included in the current study only enclosed the subscale of persistence. It would be interesting to find out whether similar relations can be obtained for the impulsivity component of effortful control. We believe this investigation is especially relevant in light of the seemingly deliberate problem talk in boys (i.e., boys high in EC co-ruminating more) and the lack of similar findings in girls. It is possible that the impulsive component of EC is more relevant in girls. 

## 5. Conclusions

The current study increased insight into potential factors that make adolescents more prone to co-ruminate. Consistent with previous research, girls were more likely to co-ruminate. Social reward and bonding further seemed to be important motivators to start to co-ruminate, yet at the same time, PA predicted decreases in co-rumination levels over time. High EC in boys was related to heightened excessive problem talk. Attachment anxiety and trust in the support of a parent in boys and girls and low attachment avoidance in boys were indicators of co-rumination, however, co-rumination levels seemed to decrease over time in the more securely attached boys.

## Figures and Tables

**Table 1:** Means and standard deviations for the total group and for boys and girls separately.

Variable	*M* (*SD*) All	*M* (*SD*) Girls	*M* (*SD*) Boys	*t*-Test (df)
Co-rumination T1	3.18 (0.74)	3.41 (0.65)	2.91 (0.75)	13.95 *** (1436.33) ^a^
Co-rumination T2	3.17 (0.79)	3.43 (0.69)	2.86 (0.80)	12.92 *** (1054.06) ^a^
Co-rumination T3	3.16 (0.79)	3.42 (0.69)	2.83 (0.78)	12.14 *** (815.03) ^a^
Co-rumination T4	3.21 (0.74)	3.46 (0.66)	2.88 (0.70)	12.47 *** (863)
Negative Affect T1	21.03 (6.72)	21.87 (7.10)	20.07 (6.13)	5.35 *** (1543.64) ^a^
Positive Affect T1	32.21 (6.72)	31.72 (6.65)	32.78 (6.76)	3.09 ** (1540)
Effortful Control T1	47.34 (7.69)	47.42 (7.72)	47.25 (7.66)	0.41 (1546)
Trust T1	34.75 (6.17)	34.77 (6.40)	34.73 (5.91)	0.11 (1534.25) ^a^
Attachment Anxiety T1	9.68 (5.78)	9.58 (5.94)	9.79 (5.59)	0.71 (1539)
Attachment Avoidance T1	18.48 (8.29)	18.07 (8.80)	18.95 (7.64)	2.10 * (1538.88) ^a^
Depressive Symptoms T1	9.23 (6.47)	10.00 (6.97)	8.34 (5.73)	5.16 *** (1540.85) ^a^
Brooding T1	10.56 (3.62)	11.18 (3.75)	9.86 (3.33)	7.35 *** (1545.50) ^a^

Note. T1 *=* Time 1, T2 = Time 2, T3 = Time 3; ^a^ *t*-test adjusted for unequal variances across gender; * *p* < 0.05; ** *p* < 0.01; *** *p* < 0.001.

**Table 2:** Intercorrelations among all variables at baseline.

	1.	2.	3.	4.	5.	6.	7.	8.	9.	10.	11.	12.
1. Co-rumination T1	-											
2. Co-rumination T2	0.49 ***	-										
3. Co-rumination T3	0.44 ***	0.62 ***	-									
4. Co-rumination T4	0.45 ***	0.57 ***	0.63 ***									
5. Negative Affect T1	0.13 ***	0.09 **	0.11 ***	0.13 ***	-							
6. Positive Affect T1	0.12 ***	0.10 ***	0.01	0.01	−0.09 ***	-						
7. Effortful Control T1	−0.01	0.00	0.01	−0.04	−0.40 ***	0.18 ***	-					
8. Trust T1	0.03	0.01	0.00	−0.03	−0.26 ***	0.28 ***	0.40 ***	-				
9. Attachment Anxiety T1	0.07 **	−0.03	0.04	0.05	0.29 ***	−0.16 ***	−0.30 ***	−0.52 ***	-			
10. Attachment Avoidance T1	−0.06 *	−0.07 *	−0.03	−0.03	0.23 ***	−0.24 ***	−0.37 ***	−0.66 ***	0.42 ***	-		
11. Depressive Symptoms T1	0.09 ***	0.07 *	0.07 *	0.11 ***	0.62 ***	−0.36 ***	−0.57 ***	−0.47 ***	0.40 ***	0.38 ***	-	
12. Brooding T1	0.24 ***	0.19 ***	0.17 ***	0.20 ***	0.54 ***	−0.04	−0.35 ***	−0.22 ***	0.27 ***	0.17 ***	0.49 ***	-
13. Age	0.08 **	0.10 ***	0.07 *	0.09 **	0.06 *	0.02	−0.24 ***	−0.19 ***	0.00	0.21 ***	0.12 ***	0.06 *

Note. T1 *=* Time 1, T2 = Time 2, T3 = Time 3; * *p* < 0.05; **p < 0.01; *** *p* < 0.001.

**Table 3:** Multi-level analyses predicting co-rumination trajectories as a function of the covariates and gender.

	Intercept		Slope	
	Estimate (*SE*)	*t*	Estimate (*SE*)	*t*
Predicting co-rumination intercept and slope from baseline to three years later				
Level 1				
Time	2.60 (0.15)	17.44 ***	−0.04 (0.07)	−0.49
Level 2				
Gender	0.24 (0.02)	13.76 ***	0.02 (0.01)	2.07 *
Brooding	0.15 (0.02)	7.53 ***	−0.01 (0.01)	−1.39
Age	0.00 (0.00)	3.73 ***	0.00 (0.00)	0.61
Depressive symptoms	−0.04 (0.02)	−1.95	0.01 (0.01)	1.03

Note. Brooding = the Children’s Response Styles Questionnaire—extended version (CRSQ-ext); Depressive Symptoms = the Children’s Depression Inventory (CDI). Gender: boys coded as −1 and girls coded as 1. * *p* < 0.05; *** *p* < 0.001.

**Table 4:** Multi-level analyses predicting co-rumination trajectories as a function of the covariates, gender, and temperament.

	Intercept		Slope	
	Estimate (*SE*)	*t*	Estimate (*SE*)	*t*
Predicting co-rumination intercept and slope from baseline to three years later				
Level 1				
Time	2.54 (0.13)	18.85 ***	0.01 (0.01)	1.09
Level 2				
Gender	0.24 (0.02)	13.21 ***	0.01 (0.01)	1.27
Brooding	0.13 (0.02)	6.91 ***	/	/
Age	0.00 (0.00)	4.58 ***	/	/
Depressive symptoms	0.03 (0.02)	1.39	/	/
NA	0.00 (0.02)	0.19	−0.01 (0.01)	−0.69
PA	0.11 (0.02)	6.09 ***	−0.03 (0.01)	−3.54 ***
EC	0.06 (0.02)	2.81 **	−0.02 (0.01)	−1.57
EC × NA	−0.02 (0.02)	−1.42	0.00 (0.01)	0.42
EC × PA	−0.01 (0.02)	−0.48	0.00 (0.01)	−0.25
Gender × NA	−0.04 (0.02)	−2.02 *	0.02 (0.01)	1.68
Gender × PA	0.02 (0.02)	1.33	0.01 (0.01)	0.71
Gender × EC	−0.07 (0.02)	−3.52 ***	0.02 (0.01)	1.89
Gender × EC × NA	0.00 (0.02)	0.22	−0.01 (0.01)	−0.55
Gender × EC × PA	0.00 (0.02)	−0.11	0.01 (0.01)	0.80

Note: NA and PA = Positive and Negative Affect Scale (PANAS); EC = Effortful Control Scale (ECS); Brooding = the Children’s Response Styles Questionnaire—extended version (CRSQ-ext); Depressive Symptoms = the Children’s Depression Inventory (CDI). Gender: boys coded as −1 and girls coded as 1. * *p* < 0.05; ** *p* < 0.01; *** *p* < 0.001.

**Table 5:** Multi-level analyses predicting co-rumination trajectories as a function of the covariates, gender, and trust.

	Intercept		Slope	
	Estimate (*SE*)	*t*	Estimate (*SE*)	*t*
Predicting co-rumination intercept and slope from baseline to three years later				
Level 1				
Time	2.51 (0.14)	18.58 ***	0.01 (0.01)	1.01
Level 2				
Gender	0.23 (0.02)	13.77 ***	0.02 (0.01)	1.88
Brooding	0.13 (0.02)	7.96 ***	/	/
Age	0.00 (0.00)	4.80 ***	/	/
Depressive symptoms	−0.01 (0.02)	−0.64	/	/
Trust	0.06 (0.02)	3.13 **	−0.02 (0.01)	−2.52 *
Gender × Trust	−0.03 (0.02)	−1.93	0.02 (0.01)	2.01 *

Note: Brooding = the Children’s Response Styles Questionnaire—extended version (CRSQ-ext); Depressive Symptoms = the Children’s Depression Inventory (CDI); Trust = People in My Life Questionnaire (PIML). Gender: boys coded as −1 and girls coded as 1. * *p* < 0.05; ** *p* < 0.01; *** *p* < 0.001.

**Table 6:** Multi-level analyses predicting co-rumination trajectories as a function of the covariates, gender, and anxious and avoidant attachment.

	Intercept		Slope	
	Estimate (*SE*)	*t*	Estimate (*SE*)	*t*
Predicting co-rumination intercept and slope from baseline to three years later				
Level 1				
Time	2.46 (0.14)	18.21 ***	0.01 (0.01)	0.76
Level 2				
Gender	0.23 (0.02)	13.52 ***	0.02 (0.01)	2.09 *
Brooding	0.13 (0.02)	7.76 ***	/	/
Age	0.00 (0.00)	5.19 ***	/	/
Depressive symptoms	−0.02 (0.02)	−0.94	/	/
Anxious Attachment	0.05 (0.02)	2.23 *	−0.01 (0.01)	−1.08
Avoidant Attachment	−0.10 (0.02)	−4.95 ***	0.02 (0.01)	1.92
Gender × Anxious	−0.03 (0.02)	−1.29	0.00 (0.01)	0.03
Gender × Avoidant	0.07 (0.02)	3.52 ***	−0.00 (0.01)	−0.43

Note. Brooding = the Children’s Response Styles Questionnaire—extended version (CRSQ-ext); Depressive Symptoms = the Children’s Depression Inventory (CDI); Anxious and Avoidant = Experiences in Close Relationships Scale—Revised Child version (ECR-R). Gender: boys coded as −1 and girls coded as 1. * *p* < 0.05; *** *p* < 0.001.

## Data Availability

The data presented in this study are available on request from the corresponding author due to restrictions. Ethics committee approval will need to be granted prior to sharing any data publicly.

## References

[1] Taylor S.E. (2011). Tend and befriend theory. Handbook of Theories of Social Psychology: Collection.

[2] Barasch A. (2020). The consequences of sharing. Curr. Opin. Psychol..

[3] Rose A.J. (2002). Co–Rumination in the Friendships of Girls and Boys. Child Dev..

[4] Rose A.J., Carlson W., Waller E.M. (2007). Prospective associations of co-rumination with friendship and emotional adjustment: Considering the socioemotional trade-offs of co-rumination. Dev. Psychol..

[5] Hankin B.L., Stone L., Wright P.A. (2010). Corumination, interpersonal stress generation, and internalizing symptoms: Accumulating effects and transactional influences in a multiwave study of adolescents. Dev. Psychopathol..

[6] Spendelow J.S., Simonds L.M., Avery R.E. (2017). The Relationship between Co-rumination and Internalizing Problems: A Systematic Review and Meta-analysis. Clin. Psychol. Psychother..

[7] Balsamo M., Carlucci L., Sergi M.R., Murdock K.K., Saggino A. (2015). The Mediating Role of Early Maladaptive Schemas in the Relation between Co-Rumination and Depression in Young Adults. PLoS ONE.

[8] Carlucci L., D’Ambrosio I., Innamorati M., Saggino A., Balsamo M. (2018). Co-rumination, anxiety, and maladaptive cognitive schemas: When friendship can hurt. Psychol. Res. Behav. Manag..

[9] Dirghangi S., Kahn G., Laursen B., Brendgen M., Vitaro F., Dionne G., Boivin M. (2015). Co-rumination cultivates anxiety: A genetically informed study of friend influence during early adolescence. Dev. Psychol..

[10] Stone L.B., Uhrlass D.J., Gibb B.E. (2010). Co-rumination and Lifetime History of Depressive Disorders in Children. J. Clin. Child Adolesc. Psychol..

[11] Stone L.B., Hankin B.L., Gibb B.E., Abela J.R.Z. (2011). Co-rumination predicts the onset of depressive disorders during adolescence. J. Abnorm. Psychol..

[12] Rose A.J., Glick G.C., Smith R.L., Schwartz-Mette R.A., Borowski S.K. (2017). Co-Rumination Exacerbates Stress Generation among Adolescents with Depressive Symptoms. J. Abnorm. Child Psychol..

[13] Stone L.B., Silk J.S., Oppenheimer C.W., Allen K.B., Waller J.M., Dahl R.E. (2017). Linking Maternal Socialization of Emotion Regulation to Adolescents’ Co-rumination With Peers. J. Early Adolesc..

[14] Rubin K.H., Bukowski W.M., Bowker J.C. (2015). Children in Peer Groups. Handbook of Child Psychology and Developmental Science.

[15] Rothbart M.K., Bates J.E., Damon W., Eisenberg N. (1998). Temperament. Handbook of Child Psychology: Social, Emotional and Personality Development.

[16] Derryberry D., Rothbart M.K. (1997). Reactive and effortful processes in the organization of temperament. Dev. Psychopathol..

[17] Belsky J., Belsky J., Hsieh K.-H., Crnic K. (1996). Infant positive and negative emotionality: One dimension or two?. Dev. Psychol..

[18] Hudson M.R., Harding K.A., Mezulis A. (2015). Dampening and brooding jointly link temperament with depressive symptoms: A prospective study. Pers. Individ. Differ..

[19] Mezulis A.H., Rudolph M.E. (2012). Pathways linking temperament and depressive symptoms: A short-term prospective diary study among adolescents. Cogn. Emot..

[20] Verstraeten K., Bijttebier P., Vasey M., Raes F. (2011). Specificity of worry and rumination in the development of anxiety and depressive symptoms in children. Br. J. Clin. Psychol..

[21] Mezulis A.H., Priess H.A., Hyde J.S. (2010). Rumination Mediates the Relationship between Infant Temperament and Adolescent Depressive Symptoms. Depress. Res. Treat..

[22] Ramsey M.A., Gentzler A.L. (2015). An upward spiral: Bidirectional associations between positive affect and positive aspects of close relationships across the life span. Dev. Rev..

[23] Yu J., Hu P.J.-H., Cheng T.-H. (2015). Role of Affect in Self-Disclosure on Social Network Websites: A Test of Two Competing Models. J. Manag. Inf. Syst..

[24] Spoor J.R., Kelly J.R. (2004). The evolutionary significance of affect in groups: Communication and group bonding. Group Process. Intergroup Relat..

[25] Forgas J.P. (2011). Affective influences on self-disclosure: Mood effects on the intimacy and reciprocity of disclosing personal information. J. Pers. Soc. Psychol..

[26] Rothbart M.K., Kohnstamm G.A., Bates J.E., Rothbart M.K. (1989). Temperament and development. Temperament in Childhood.

[27] Verstraeten K., Vasey M., Raes F., Bijttebier P. (2008). Temperament and Risk for Depressive Symptoms in Adolescence: Mediation by Rumination and Moderation by Effortful Control. J. Abnorm. Child Psychol..

[28] Bowlby J. (1969). Attachment and Loss: Vol. 1. Attachment.

[29] Shaver P.R., Mikulincer M. (2002). Attachment-related psychodynamics. Attach. Hum. Dev..

[30] Kobak R.R., Sceery A. (1988). Attachment in Late Adolescence: Working Models, Affect Regulation, and Representations of Self and Others. Child Dev..

[31] Ainsworth M.D., Blehar M.C., Waters E., Wall S. (1978). Patterns of Attachment.

[32] Cassidy J. (1994). Emotion regulation: Influences of attachment relationships. Monogr. Soc. Res. Child Dev..

[33] Fraley R.C., Spieker S.J. (2003). Are infant attachment patterns continuously or categorically distributed? A taxometric analysis of strange situation behavior. Dev. Psychol..

[34] Bowlby J. (1980). Attachment and Loss: Vol. 3. Loss: Sadness and Depression.

[35] Mikulincer M., Shaver P. (2007). Attachment in Adulthood: Structure, Dynamics, and Change.

[36] Mikulincer M., Shaver P.R., Pereg D. (2003). Attachment theory and affect regulation: The dynamics, development, and cognitive consequences of attachment-related strategies. Motiv. Emot..

[37] Campbell L., Simpson J.A., Boldry J., Kashy D.A. (2005). Perceptions of Conflict and Support in Romantic Relationships: The Role of Attachment Anxiety. J. Pers. Soc. Psychol..

[38] Van de Walle M., Bijttebier P., Braet C., Bosmans G. (2016). Attachment Anxiety and Depressive Symptoms in Middle Childhood: The Role of Repetitive Thinking about Negative Affect and about Mother. J. Psychopathol. Behav. Assess..

[39] Pearson K.A., Watkins E.R., Mullan E.G., Moberly N.J. (2010). Psychosocial correlates of depressive rumination. Behav. Res. Ther..

[40] Brenning K., Soenens B., Braet C., Bosmans G. (2011). The Role of Depressogenic Personality and Attachment in the Intergenerational Similarity of Depressive Symptoms: A Study with Early Adolescents and Their Mothers. Pers. Soc. Psychol. Bull..

[41] Lanciano T., Curci A., Kafetsios K., Elia L., Zammuner V.L. (2012). Attachment and dysfunctional rumination: The mediating role of Emotional Intelligence abilities. Pers. Individ. Differ..

[42] Reynolds S., Searight H.R., Ratwik S. (2014). Adult attachment styles and rumination in the context of intimate relationships. N. Am. J. Psychol..

[43] Cox S.J., Mezulis A.H., Hyde J.S. (2010). The influence of child gender role and maternal feedback to child stress on the emergence of the gender difference in depressive rumination in adolescence. Dev. Psychol..

[44] Chaplin T.M. (2015). Gender and Emotion Expression: A Developmental Contextual Perspective. Emot. Rev..

[45] Chaplin T.M., Cole P.M., Zahn-Waxler C. (2005). Parental Socialization of Emotion Expression: Gender Differences and Relations to Child Adjustment. Emotion.

[46] Brody L.R., Fischer A.H. (2000). The socialization of gender differences in emotional expression: Display rules, infant temperament, and differentiation. Gender and Emotion: Social Psychological Perspectives.

[47] Klimes-Dougan B., Brand A.E., Zahn-Waxler C., Usher B., Hastings P.D., Kendziora K., Garside R.B. (2007). Parental Emotion Socialization in Adolescence: Differences in Sex, Age and Problem Status. Soc. Dev..

[48] Velotti P., D’Aguanno M., De Campora G., Di Francescantonio S., Garofalo C., Giromini L., Petrocchi C., Terrasi M., Zavattini G.C. (2015). Gender moderates the relationship between attachment insecurities and emotion dysregulation. S. Afr. J. Psychol..

[49] Mikulincer M., Shaver P.R. (2007). Boosting Attachment Security to Promote Mental Health, Prosocial Values, and Inter-Group Tolerance. Psychol. Inq..

[50] Timbremont B., Braet C., Roelofs J. (2008). Handleiding Children’s Depression Inventory (Herziene Versie). Manual for Children’s Depression Inventory (Revised Version).

[51] Watson D., Clark L.A., Tellegen A. (1988). Development and validation of brief measures of positive and negative affect: The PANAS scales. J. Pers. Soc. Psychol..

[52] Van Wijk C.M.T., Kolk A.M. (1996). Psychometric evaluation of symptom perception related measures. Personal. Individ. Differ..

[53] Lonigan C.J., Phillips B.M., Dadds M.R. (2001). Temperamental Influences on the Development of Anxiety Disorders. The Developmental Psychopathology of Anxiety.

[54] Verstraeten K., Vasey M.W., Claes L., Bijttebier P. (2010). The assessment of effortful control in childhood: Questionnaires and the Test of Everyday Attention for Children compared. Pers. Individ. Differ..

[55] Eisenberg N., Sadovsky A., Spinrad T.L., Fabes R.A., Losoya S.H., Valiente C., Reiser M., Cumberland A., Shepard S.A. (2005). The relations of problem behavior status to children’s negative emotionality, effortful control, and impulsivity: Concurrent relations and prediction of change. Dev. Psychol..

[56] Ridenour T., Greenberg M.T., Cook E.T. (2006). Structure and validity of people in my life: A self-report measure of attachment in late childhood. J. Youth Adolesc..

[57] Bosmans G., Braet C., Heylen J., De Raedt R. (2015). Children’s Attentional Processing of Mother and Proximity Seeking. PLoS ONE.

[58] Fraley R.C., Waller N.G., Brennan K.A. (2000). An item response theory analysis of self-report measures of adult attachment. J. Pers. Soc. Psychol..

[59] Brenning K., Soenens B., Braet C., Bosmans G. (2011). An adaptation of the Experiences in Close Relationships Scale-Revised for use with children and adolescents. J. Soc. Pers. Relatsh..

[60] Kovacs M. (2003). Multi-health Systems. Children’s Depression Inventory: Technical Manual.

[61] Abela J.R.Z., Brozina K., Haigh E.P. (2002). An Examination of the Response Styles Theory of Depression in Third- and Seventh-Grade Children: A Short-Term Longitudinal Study. J. Abnorm. Child Psychol..

[62] Hedeker D., Gibbons R.D. (2006). Longitudinal Data Analysis.

[63] Groh A.M., Narayan A.J., Bakermans-Kranenburg M.J., Roisman G.I., Vaughn B.E., Fearon R.M.P., Ijzendoorn M.H. (2017). Attachment and Temperament in the Early Life Course: A Meta-Analytic Review. Child Dev..

[64] Isen A.M. (2008). Some ways in which positive affect influences decision making and problem solving. Handbook of Emotions.

[65] Byrd-Craven J., Granger D.A., Auer B.J. (2011). Stress reactivity to co-rumination in young women’s friendships: Cortisol, alpha-amylase, and negative affect focus. J. Soc. Pers. Relatsh..

[66] Broderick P.C. (1998). Early Adolescent Gender Differences in the Use of Ruminative and Distracting Coping Strategies. J. Early Adolesc..

[67] Bastin M., Mezulis A.H., Ahles J., Raes F., Bijttebier P. (2015). Moderating Effects of Brooding and Co-Rumination on the Relationship Between Stress and Depressive Symptoms in Early Adolescence: A Multi-Wave Study. J. Abnorm. Child Psychol..

[68] Haggard D.L., Robert C., Rose A.J. (2011). Co-Rumination in the Workplace: Adjustment Trade-offs for Men and Women Who Engage in Excessive Discussions of Workplace Problems. J. Bus. Psychol..

[69] Barnett A.G., van der Pols J., Dobson A.J. (2004). Regression to the mean: What it is and how to deal with it. Int. J. Epidemiol..

[70] Zahn–Waxler C., Klimes-Dougan B., Slattery M.J. (2000). Internalizing problems of childhood and adolescence: Prospects, pitfalls, and progress in understanding the development of anxiety and depression. Dev. Psychopathol..

[71] Bastin M., Vanhalst J., Raes F., Bijttebier P. (2018). Co-Brooding and Co-Reflection as Differential Predictors of Depressive Symptoms and Friendship Quality in Adolescents: Investigating the Moderating Role of Gender. J. Youth Adolesc..

[72] Bastin M., Bijttebier P., Raes F., Vasey M.W. (2014). Brooding and reflecting in an interpersonal context. Pers. Individ. Differ..

[73] Waller E.M., Rose A.J. (2013). Brief report: Adolescents’ co-rumination with mothers, co-rumination with friends, and internalizing symptoms. J. Adolesc..

[74] Brand A.E., Klimes-Dougan B. (2010). Emotion socialization in adolescence: The roles of mothers and fathers. New Dir. Child Adolesc. Dev..

[75] Stone L.B., Gibb B.E. (2015). Brief report: Preliminary evidence that co-rumination fosters adolescents’ depression risk by increasing rumination. J. Adolesc..

[76] Bastin M., Luyckx K., Raes F., Bijttebier P. (2021). Co-Rumination and Depressive Symptoms in Adolescence: Prospective Associations and the Mediating Role of Brooding Rumination. J. Youth Adolesc..

[77] Jose P.E., Wilkins H., Spendelow J.S. (2012). Does social anxiety predict rumination and co-rumination among adolescents?. J. Clin. Child Adolesc. Psychol..

[78] Davis M., Suveg C. (2014). Focusing on the Positive: A Review of the Role of Child Positive Affect in Developmental Psychopathology. Clin. Child Fam. Psychol. Rev..

[79] Hamilton C.E. (2000). Continuity and Discontinuity of Attachment from Infancy through Adolescence. Child Dev..

